# Changes of Dry Eye Related Markers and Tear Inflammatory Cytokines After Upper Blepharoplasty

**DOI:** 10.3389/fmed.2021.763611

**Published:** 2021-12-09

**Authors:** Songjiao Zhao, Nan Song, Lan Gong

**Affiliations:** ^1^Department of Ophthalmology, Eye, Ear, Nose and Throat Hospital of Fudan University, Shanghai, China; ^2^Department of Facial Plastic and Reconstructive Surgery, Eye, Ear, Nose and Throat Hospital of Fudan University, Shanghai, China; ^3^Myopia Key Laboratory of Ministry of Health, Eye, Ear, Nose and Throat Hospital of Fudan University, Shanghai, China

**Keywords:** dry eye, ocular surface, tear inflammatory cytokines, postoperative, upper blepharoplasty

## Abstract

**Objectives:** To investigate the changes of dry eye-related clinical manifestations, ocular surface parameters, and tear inflammatory cytokines after upper blepharoplasty.

**Methods:** Forty eyes of 20 who underwent upper blepharoplasty were divided into either the group with or the group without preexisting dry eye before upper blepharoplasty. Ocular Surface Disease Index (OSDI), Schirmer I test, tear meniscus height, lipid layer thickness, non-invasive tear break-up time (NIKBUT), fluorescein tear film break-up time (FBUT), corneal fluorescein staining, meibum expression, lid margin changes, and tear inflammatory cytokines were assessed preoperatively and at 1, 3, and 6 months postoperatively. Correlations between inflammatory cytokines and dry eye-related parameters were determined.

**Results:** The OSDI scores increased significantly at 1 month (*p* = 0.040) and subsequently decreased to the preoperative levels at 6 months postoperatively in subjects with dry eye. First (f)-NIKBUT and FBUT were significantly shortened at 1, 3, and 6 months postoperatively in subjects with dry eye (f-NIKBUT: *p* <0.001, *p* = 0.010, *p* = 0.042; FBUT: *p* = 0.002, *p* = 0.005, *p* = 0.037, respectively), but were only shortened at 1 month (*p* = 0.028, *p* = 0.005) and returned to baseline levels at 6 months postoperatively in subjects without preexisting dry eye. A significant increasing trend of interleukin (IL)-6 was found in both dry eye and subjects without preexisting dry eye (*p* = 0.016, *p* = 0.008), while IL-8 and tumor necrosis factor alpha (TNF-α) were only found to be increased in subjects with dry eye postoperatively (*p* = 0.031, *p* = 0.031). The levels of IL-8 and TNF-α were positively correlated with OSDI scores (*p* = 0.046, *p* = 0.043, respectively) and negatively correlated with f-NIKBUT and FBUT (*p* = 0.026, *p* = 0.006, respectively).

**Conclusions:** Upper blepharoplasty might increase the release of tear inflammatory cytokines and tear film instability that contribute to the development of postoperative dry eye in the early postoperative period and the changes most relieved in 6 months. Preexisting dry eye is a higher risk factor for worse and persistent ocular surface damage after upper blepharoplasty.

## Introduction

Upper blepharoplasty is one of the most popular esthetic surgeries in Asia to create an upper eyelid crease and improve appearance and functional visual field (1). This procedure generally involves the removal of redundant upper eyelid skin, orbital fat, and/or the orbicularis oculi muscle to achieve periorbital rejuvenation (2). Changes in upper eyelid anatomy and movement dynamics may be attributed to the development of dry eye disease after upper blepharoplasty, with a reported incidence of 0–12.9% (3–5). Dry eye is a multifactorial disease of the ocular surface in which tear film instability and hyperosmolarity, ocular surface inflammation and damage, and neurosensory abnormalities play etiological roles (6). Typical symptoms of postoperative dry eye are similar to those of common dry eye, including dryness, redness, burning, foreign body sensation, and occasional blurred vision (7). Although dry eye is considered a relatively rare, mild, and transient complication of upper blepharoplasty, the severity of dry eye symptoms could affect psychosomatic symptoms and quality of life (8, 9). In addition, few cases can develop into chronic, persistent dry eye syndrome, which severely affects the physical and mental health of patients (3). To prevent and manage dry eye after upper blepharoplasty, investigating the potential pathogenesis *via* preoperative and postoperative evaluation of the ocular surface is pivotal.

Postoperative dry eye triggered by upper blepharoplasty is generally considered to be related to the decline of tear film stability. Several potential mechanisms contributing to this phenomenon have been proposed: the uneven distribution of tear film caused by the change in interaction between the eyelid and ocular surface (10); decreased lipid secretion and increased tear evaporation induced by the reduced blink rate and incomplete blinking; and postoperative lagophthalmos (3, 11–13). However, a recent study showed that palpebral aperture or blink dynamics changes are not the main cause of dry eye following upper blepharoplasty (14). We wondered whether there are other factors associated with the development of this complication. The updated definition of dry eye specifies the critical role of ocular surface inflammation in the course of the disease (6). Several studies identified an inflammatory cascade that may occur following ocular and intraocular surgeries, such as LASIK and cataract surgery, which lead to direct damage of the ocular surface (15, 16). Another recent study showed that frontalis suspension surgery resulted in the elevation of inflammatory cytokine levels on the ocular surface in the early period postoperative (17). Existing research on dry eye after upper blepharoplasty focused on tear film stability and tear fluid dynamics. However, changes in ocular surface inflammation after upper blepharoplasty have not been reported.

In this study, we evaluated the changes in dry eye-related markers and tear inflammatory cytokines in patients who underwent upper blepharoplasty. Furthermore, the correlations between inflammatory cytokines and dry eye-related markers were determined. We aimed to characterize the effects of upper blepharoplasty on the ocular surface microenvironment and investigate the potentially novel pathogenesis of postoperative dry eye following upper blepharoplasty.

## Materials and Methods

### Patient Characteristics

Twenty patients (19 women, 1 man; age range, 21–35 years) who underwent bilateral upper blepharoplasty at the Eye, Ear, Nose, and Throat Hospital of Fudan University from August 2020 to January 2021 were recruited for this prospective study. The study was conducted in accordance with the World Medical Association Declaration of Helsinki. The study was approved by the ethics committee of the Eye, Ear, Nose, and Throat Hospital of Fudan University (number 2019093-1), and written informed consent was obtained from all patients who participated.

All surgical candidates underwent a thorough ocular surface assessment by an ophthalmologist before surgery and at 1, 3, and 6 months after surgery. The candidates were divided into two groups according to whether they had preexisting dry eye disease. The diagnostic criteria for dry eye disease included the presence of at least one of the following symptoms, dryness, burning sensation, grittiness, photophobia, pain, and tickle, and one of the following signs: Schirmer I test ≤ 5 mm/5 min; tear film break-up time <5 s; positive fluorescein staining with either 5 mm/5 min < Schirmer I test ≤ 10 mm/5 min or 5 s ≤ tear film break-up time <10 s. Eighteen eyes of nine enrolled subjects met the criteria for dry eye and were classified as the dry eye group. The remaining 22 eyes of 11 patients were in the group without preexisting dry eye. The exclusion criteria included a history of eyelid surgery, a history of ocular surgery within 6 months, neuromuscular abnormalities, a history of wearing contact lenses, ocular/eyelid diseases such as acute inflammation or infection, glaucoma, lagophthalmos; systemic diseases that would probably affect the ocular surface, such as Sjögren's syndrome; and inability to follow or cooperate during the examinations.

### Surgical Techniques

All subjects underwent cosmetic, bilateral upper blepharoplasty by the same senior oculoplastic surgeon (Nan Song) under topical anesthesia with 2% lidocaine. In brief, the operation process involves the following steps: excision of excess upper eyelid skin and a strip of preseptal orbicularis oculi muscle, removal of excess fat, and exposure of septoaponeurosis junctional thickening, which is stitched with the lower edge orbicularis oculi muscle using 6-0 non-absorbable suture, and the eyelid is closed with 7-0 absorbable suture. No anti-inflammatory eye drops were used after surgery.

### Ocular Surface Evaluation

All subjects underwent a thorough ocular examination with the help of an ophthalmologist in the following conditions: the interval between the tests was >15 min; the examination room was quiet, with a constant temperature of 25°C and 50% humidity; and none of the patients used any eye drops on the day of the examination.

#### Ocular Surface Disease Index

Dry eye symptoms were assessed using the OSDI questionnaire. Twelve questions were included to quantify the discomfort in a 1-week period, covering three subscales: ocular symptoms, vision-related function, and environmental stimulant. The OSDI score ranges from 0 to 100, and a higher OSDI score indicates greater disability.

#### Non-invasive Tear Break-Up Time and Tear Meniscus Height

NIKBUT and TMH were measured using an OCULUS Keratograph 5M (Wetzlar, Germany) equipped with modified TF-scan software. All subjects were requested to blink twice and then keep their eyes open as much time as possible until the next blink. The outputs included the first non-invasive tear break-up time (f-NIKBUT) and the average non-invasive tear break-up time (av-NIKBUT). The tear meniscus height was measured three times, as previously reported (18) using infrared images taken at the central point of the lower lid margin. The procedure was repeated three times for each eye in a dark room.

#### Lipid Layer Thickness

Lipid layer thickness was measured non-invasively by tear interferometry using a LipiView instrument (TearScience, Morrisville, NC, United States). All subjects were instructed to blink naturally to record a 15 s video of the tear film interference pattern and analyze the thickness of the lipid layer. The procedure was repeated three times for each eye.

#### Fluorescein Tear Film Break-Up Time

After instilling fluorescein into the lower fornix using fluorescein strips (Jingming, Tianjing, China), the subjects were asked to blink naturally several times and then keep their eyes open for as long as possible until the next blink. The time between the last blink and the occurrence of the first dry spot on the cornea was observed under cobalt blue light and recorded as FBUT. The procedure was repeated three times for each eye.

#### Corneal Fluorescein Staining

After measuring the FBUT, corneal FL was performed. To quantify punctate staining, the corneal surface was divided into five areas, as proposed by the US National Eye Institute (19). The grade of punctate staining in each area was recorded as 0–3, 0, no staining; 1, <15 dots; 2, 16 −20 dots; 3, > 30 dots, strip/bulk staining, or corneal filaments. The total FL scores ranged from 0 to 15.

#### Schirmer I Test

The Schirmer test without anesthesia (Schirmer I test) was used to assess basic tears. A 40 mm Schirmer paper strip (Jingming, Tianjing, China) was placed into the temporal one-third portion of the lower conjunctival fornix. The subjects were asked to close their eyes gently and sit quietly for 5 min. The length of the wetting part from the folded notch was recorded.

#### Meibomian Gland Evaluation

Evaluation of meibomian gland function was performed under slit lamp using a grading method using meibum expression and lid margin changes (20). The upper eyelid was digitally pressed and the meibum expression was graded based on the expressibility and meibum quality as 0–2: 0, clear and normal volume; 1, opaque with hyperviscosity and/or reduced; and 2, not expressed. Lid margin changes were graded based on the irregularity of the lid margin, telangiectasia, plugging of meibomian orifices, and replacement of the mucocutaneous junction, ranging from 0 to 4 (20).

### Tear Inflammation Cytokines

#### Tear Sample Collection

Disposable 2.2 μL tear collectors (Seinda, Guangdong, China) were used to obtain tear fluid at the lateral canthus before surgery and at 1, 3, and 6 months postoperatively. A total sample of 15 μL without being diluted was collected without anesthesia or irritation of the cornea, conjunctiva, or lid margin. Tear samples were transferred into 0.5 mL microtubes (Axygen, Union City, United States), immediately placed on ice, and then stored at −80°C for further experiments.

#### Assays for Tear Inflammation Cytokines

Luminex liquid suspension chip detection was performed using Wayen Biotechnologies (Shanghai, China). MILLIPLEX MAP High Sensitivity T Cell Magnetic Bead Panel (Merck EMD Millipore, Billerica, MA, United States) for interleukin (IL)-1β, IL-6, IL-8, tumor necrosis factor alpha (TNF-α), and interferon-gamma (IFN-γ) was used according to the manufacturer's instructions. Briefly, tear samples were incubated in microbead-embedded 96-well plates overnight at 4°C and subsequently incubated with detection antibody for 1 h at room temperature. Next, streptavidin-phycoerythrin was added into each well of the plate and incubated for 30 min at room temperature, and the values were read using a Luminex 200 system (Luminex Corporation, Austin, TX, United States).

### Statistical Analyses

**Figure 1:**
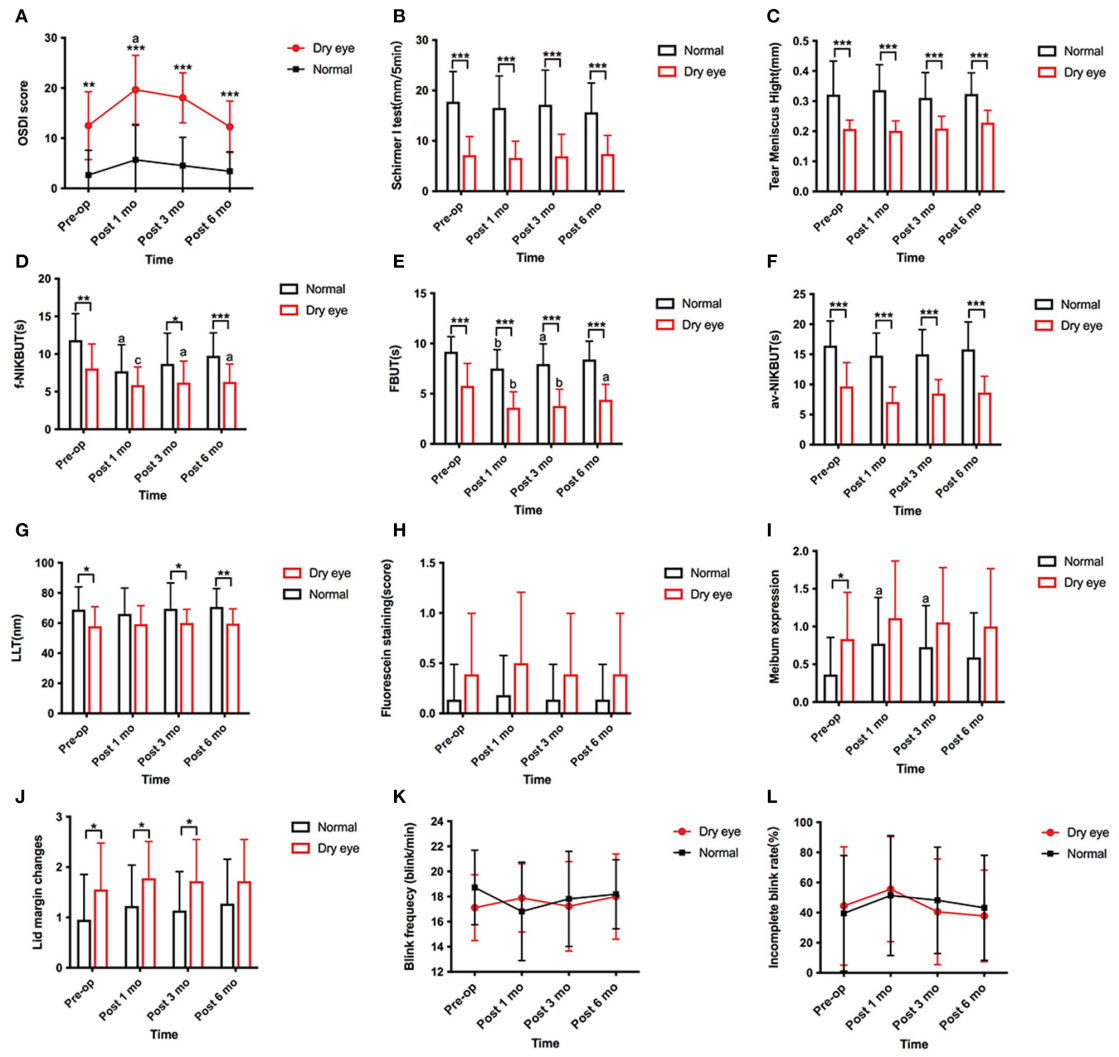
Changes in dry eye related parameters preoperatively, 1, 3, and 6 months postoperatively from preoperative value. **(A)** Change in the Ocular Surface Disease Index (OSDI) score. The OSDI score is significantly increased in subjects with dry eye at 1 month postoperatively (*p* = 0.040) and subsequently decreased to preoperative level at 6 months postoperatively. No significant change shows in subjects without dry eye from preoperative value. Subjects with dry eye have higher OSDI scores at any follow up time than normal (*p* < 0.001). **(B,C)** Changes in Schirmer I test and tear meniscus height (TMH). No significant difference is found in Schirmer I test or TMH at any follow up time in both subject with and without dry eye. Subjects with dry eye show a less tear production at any follow up time than normal (*p* < 0.001). **(D)** Changes in first non-invasive tear break-up time (f-NIKBUT) from preoperative value. f-NIKBUT in dry eye subjects is significantly shortened at 1, 3, and 6 months postoperatively (*p* < 0.001, *p* = 0.010, *p* = 0.042). In subjects without dry eye, the f-NIKBUT is significantly shortened at 1 month postoperatively (*p* = 0.028) and returns to baseline levels at 6 months postoperatively. Subjects with dry eye shows a decreased f-NIKBUT than normal at 3 and 6 months postoperatively (*p* = 0.037, *p* < 0.001). **(E)** Changes in fluorescein tear film break-up time (FBUT) from preoperative value. FBUT in subjects with dry eye is significantly shortened at 1, 3, and 6 months postoperatively compared with the preoperative measurements (*p* = 0.002, *p* = 0.005, *p* = 0.037). FBUT is significantly shortened at 1 month postoperatively (*p* = 0.005) and returns to baseline levels at 6 months postoperatively in subjects without dry eye. **(F–H)** Changes in average non-invasive tear break-up time (av-NIKBUT), lipid layer thickness (LLT) and corneal fluorescein staining from preoperative values. No significant changes are found in av-NIKBUT, LLT and fluorescein staining scores at any follow up time postoperatively. **(I,J)** Changes in meibum expression and lid margin changes. Meibum expression scores are significantly higher in normal subjects at 1 and 3 months postoperatively (*p* = 0.012, *p* = 0.016), while no significant change is found in subjects with dry eye. Lid margin changes show no significant change at any follow up time in subjects with and without dry eye. **(K,L)** Changes in blink frequency and incomplete blink rate. No significant changes are shown in blink frequency and incomplete blink rate at any follow up time in subjects with and without dry eye (*p < 0.05, **p < 0.01, ***p < 0.001). a, b, c: significant differences between preoperative and postoperative values. ^a^*p* < 0.05, ^b^*p* < 0.01, ^c^*p* < 0.001.

**Figure 2:**
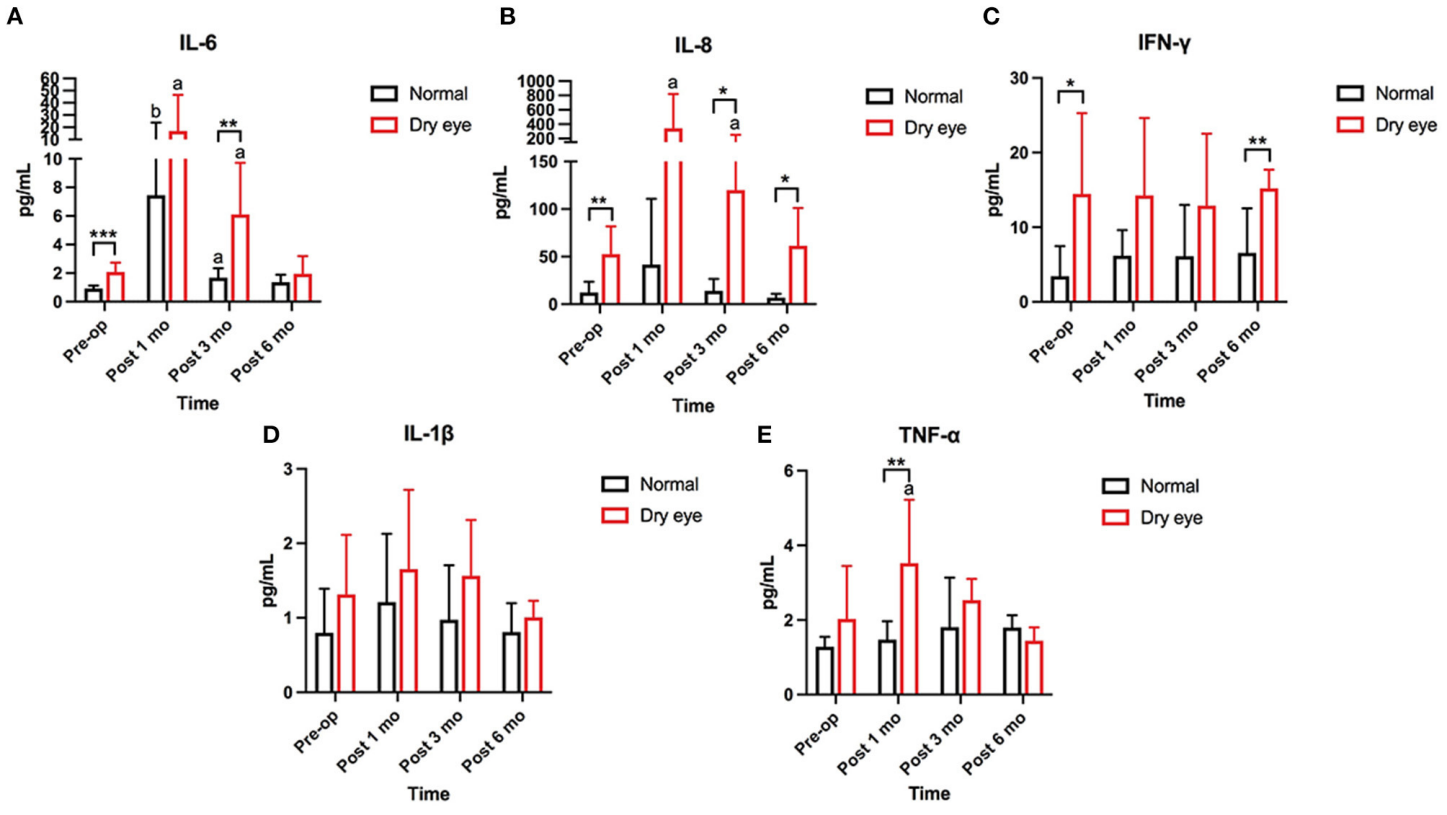
Changes in concentration of tear inflammatory cytokines from the preoperative value. **(A,B)** The baseline levels of IL-6, IL-8, and IFN-γ are significantly higher in subjects with dry eye than in subjects without dry eye (*p* < 0.001, *p* = 0.003). Increasing trends of IL-6 and IL-8 are found at 1 (*p* = 0.016, *p* = 0.031) and 3 months (*p* = 0.016, *p* = 0.047) postoperatively in subjects with dry eye. At 6 months postoperatively, the levels of IL-6 and IL-8 are gently declined but still higher than the baseline levels (*p* = 0.004, *p* = 0.047). In subjects without dry eye, only IL-6 has significantly increased at 1 and 3 months postoperatively (*p* = 0.008, *p* = 0.016). **(C)** The concentration of interferon-gamma (IFN-γ) is significantly higher in dry eye subjects than in normal subjects preoperatively and 6 months postoperatively (*p* = 0.019, *p* = 0.003). No significant change is found in IFN-γ from preoperative levels in subjects with and without dry eye. **(D)** The concentration of interleukin (IL)-1β shows no significant changes at preoperative and any follow up time in subjects with and without dry eye. **(E)** The concentration of tumor necrosis factor alpha (TNF-α) is significantly increased at 1 month (*p* = 0.031) and returns to the baseline level at 3 months in subjects with dry eye. No significant change is found in subjects without dry eye (*significant difference between subjects with and without dry eye. *p < 0.05, **p < 0.01, ***p < 0.001; a, b: significant differences between preoperative and postoperative values. ^a^*p* < 0.05, ^b^*p* < 0.01).

**Figure 3:**
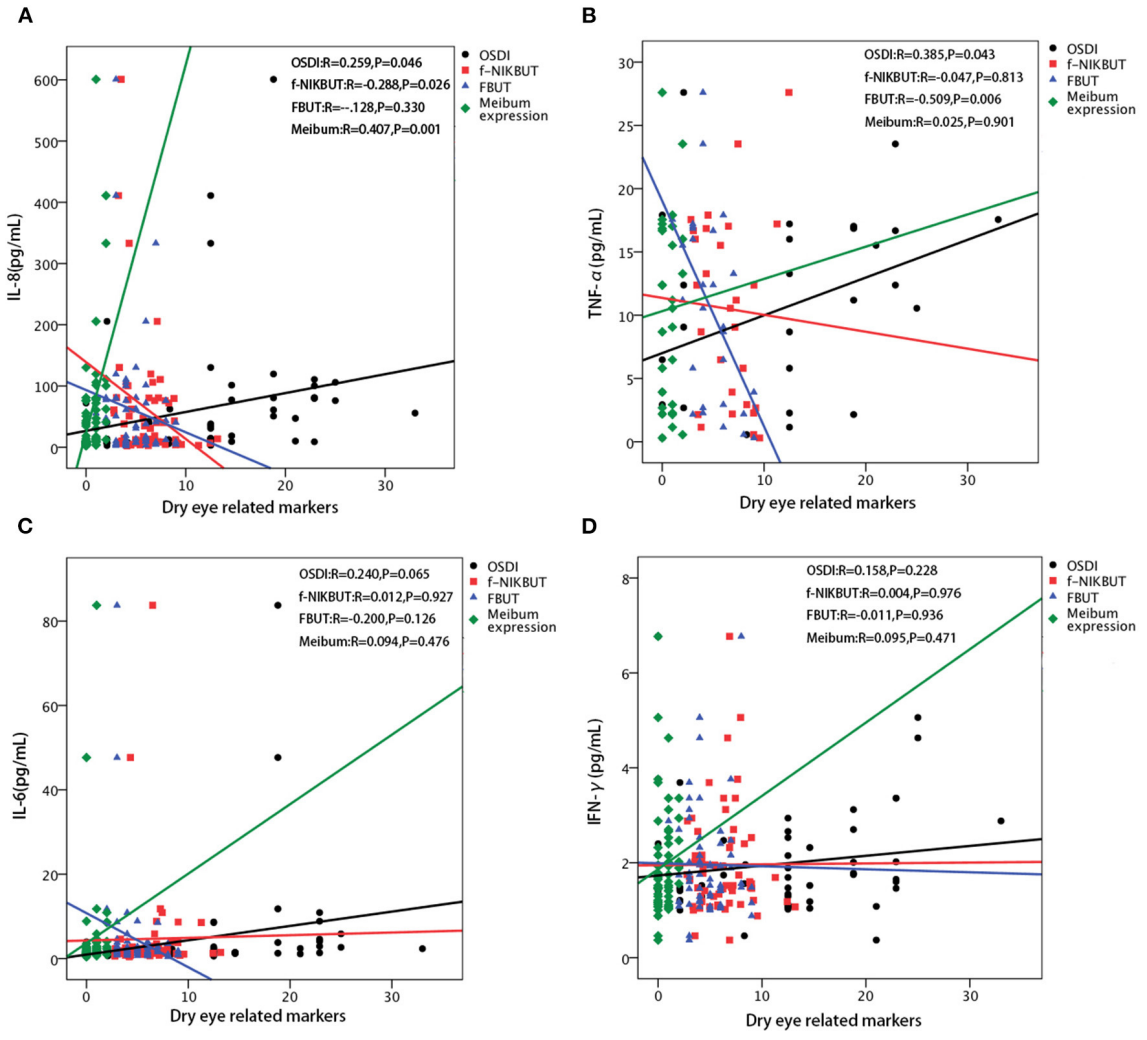
Correlations between each inflammatory cytokines and dry eye related parameters. **(A)** The level of interleukin (IL)-8 is positively correlated with the Ocular Surface Disease Index (OSDI) score (*r* = 0.259, *p* = 0.046) and meibum expression score (*r* = 0.407, *p* = 0.001), while negatively correlated with first non-invasive tear break-up time (f-NIKBUT) (*r* = −0.228, *p* = 0.026). No significant correlation is found between IL-8 and fluorescein tear film break-up time (FBUT). **(B)** The level of tumor necrosis factor alpha (TNF-α) is positively correlated with the OSDI score (*r* = 0.385, *p* = 0.043), while negatively correlated with FBUT (*r* = −0.509, *p* = 0.006). No significant correlations are found between TNF-α and f-NIKBUT, and meibum expression score. **(C)** The level of IL-6 is not significantly correlated with the OSDI score, f-NIKBUT, FBUT, and meibum expression score. **(D)** The level of interferon-gamma (IFN-γ) is not significantly correlated with OSDI, f-NIKBUT, FBUT and meibum expression score.

Data analyses were performed using SPSS 24.0 (IBM Corp., Armonk, NY, United States). The tabulated results are expressed as the mean ± standard deviation. The generalized estimating equation (21) was used to adjust the correlation between the right and left eyes and to compare the preoperative examination values between the group without and the group with dry eye (Table 1). The differences between preoperative and postoperative OSDI scores, TMH, NIKBUT, FBUT, FL score, Schirmer I test, and tear inflammatory cytokines were compared using Wilcoxon signed-rank test (Figures 1, 2). The correlations between measurements were performed using the Spearman rank correlation test (Figure 3). Statistical significance was set at *p* < 0.05.

**Table 1:** Demographic data and clinical characteristics of normal and dry eye.

	**Normal (*n* = 22)**	**Dry eye (*n* = 18)**	***P*-value**
Age	25.64 ± 1.44	28.11 ± 1.31	0.229
Sex	10F/1M	9F	
OSDI score	2.66 ± 1.49	12.51 ± 2.25	**0.001**
Schirmer I test (mm/5 min)	18.09 ± 1.19	7.17 ± 0.87	**<0.001**
TMH(mm)	0.32 ± 0.02	0.21 ± 0.01	**<0.001**
FBUT(s)	9.18 ± 0.32	5.78 ± 0.53	**<0.001**
f-NIKBUT(s)	11.85 ± 0.77	8.07 ± 0.77	**0.001**
Av-NIKBUT(s)	16.46 ± 0.87	9.69 ± 0.93	**<0.001**
FL score	0.14 ± 0.07	0.39 ± 0.14	0.110
Meibomian gland evaluation			
Meibum expression	0.36 ± 0.49	0.83 ± 0.62	**0.011**
Lid margin changes	0.83 ± 0.79	1.54 ± 1.01	**0.044**
LLT (nm)	71.17 ± 3.68	61.72 ± 3.65	0.082
Blink frequency (blink/min)	18.08 ± 1.04	17.00 ± 0.98	0.481
Incomplete blink rate (%)	39.55 ± 8.18	44.44 ± 9.26	0.694

*TMH, Tear meniscus height; FBUT, Fluorescein break-up time; f-NIKBUT, first-non-invasive keratograph tear film break-up time; Av-NIKBUT, average-non-invasive keratograph tear film break-up time; FL score, Fluorescein staining score; LLT, Lipid layer thickness. The bold values means significant results*.

## Results

### Demographic and Preoperative Dry Eye-Related Markers

Forty eyes of 20 subjects underwent bilateral upper blepharoplasty. Twenty-two eyes of 11 subjects without preexisting dry eye with a mean age of 26.00 ± 4.47 years (10 females, 1 male) were included and compared with 18 eyes of nine subjects who had dry eye (mean age: 27.67 ± 4.58 years; 9 females; *p* = 0.423). All the subjects were satisfied with the operation results, and no other complications such as wound infection or lagophthalmos occurred within 6 months postoperatively.

By comparing the preoperative dry eye-related markers, subjects with dry eye showed more severe clinical manifestations than subjects without dry eye. The OSDI score in subjects with dry eye disease was significantly higher than that in subjects without dry eye (*p* = 0.001). The values of the Schirmer I test, TMH, FBUT, f-NIKBUT, and av-NIKBUT in subjects with dry eye were significantly lower than those in subjects without dry eye (*p* < 0.001, *p* < 0.001, *p* < 0.001, *p* = 0.001, *p* < 0.001). The grades of meibum expression and lid margin changes were significantly higher in subjects with dry eye than in eyes without dry eye (*p* = 0.028, *p* = 0.006). The FL score, LLT, and blink dynamics showed no significant differences between subjects with and without dry eye. The demographic data and preoperative ocular surface parameters are listed in Table 1.

### The Effect of Upper Blepharoplasty on Dry Eye-Related Markers

To evaluate the effect of upper blepharoplasty on the ocular surface, the preoperative parameters were compared to those at 1, 3, and 6 months postoperatively in subjects with dry eye and subjects without dry eye, respectively.

#### Ocular Surface Disease Index

The OSDI scores significantly increased at 1 month (*p* = 0.040) and subsequently decreased to the preoperative levels at 6 months postoperatively in subjects with dry eye. No significant change was observed in the OSDI scores in subjects without dry eye from the preoperative value. Subjects with dry eye had higher OSDI scores at any follow-up time than subjects without dry eye (*p* < 0.001) (Figure 1A).

#### Tear Production

Tear production was assessed using the Schirmer I test and the TMH. Compared with the preoperative measurements, no significant difference was found in the Schirmer I test or TMH at any follow-up time in both subjects with and without dry eye. Subjects with dry eye showed less tear production at any follow-up time than subjects without dry eye (*p* < 0.001) (Figures 1B,C).

#### Tear Film Stability

Tear film stability was evaluated by NIKBUT, FBUT, and LLT. The values of f-NIKBUT and FBUT in subjects with dry eye were significantly reduced at 1, 3, and 6 months postoperatively compared with the preoperative measurements (f-NIKBUT: *p* < 0.001, *p* = 0.010, *p* = 0.042, respectively; FBUT: *p* = 0.002, *p* = 0.005, *p* = 0.037, respectively) (Figures 1D,E). In contrast to subjects with dry eye, f-NIKBUT and FBUT were significantly shortened at 1 month postoperatively (*p* = 0.028, *p* = 0.005) and returned to baseline levels at 6 months postoperatively in subjects without dry eye. No significant difference was observed in av-NIKBUT at any follow-up time in either subjects with or without dry eye compared to preoperative measurements (Figure 1F). A shorter tear film break-up time was demonstrated in subjects with dry eye than in subjects without dry eye at any postoperative follow-up time (*p* < 0.001). The lipid layer plays a critical role in preventing excessive evaporation and stabilizing the tear film surface. Subjects with dry eye had a thinner lipid layer than eyes without dry eye at 3 and 6 months postoperatively (*p* = 0.042, *p* = 0.003). However, the LLT values did not significantly change with time in both subjects with and without dry eye (Figure 1G).

#### Corneal Epithelium Injury

Corneal epithelium injury was quantified using the FL score. The FL scores showed no significant change at any follow-up time after surgery in both subjects with and without dry eye (Figure 1H).

#### Meibomian Gland Function

The meibomian gland function was evaluated using meibum expression and lid margin changes. In comparison with the preoperative parameters, meibum expression scores were significantly higher in subjects without dry eye at 1 and 3 months postoperatively (*p* = 0.012, *p* = 0.016), but remained unchanged in subjects with dry eye (Figure 1I). No remarkable change was found in the lid margin after surgery in both subjects with and without dry eye (Figure 1J).

#### Blink Dynamics

Blink dynamics were assessed using blink frequency and incomplete blink rate. No significant changing trend was found in the blink frequency or incomplete blink rate following upper blepharoplasty (Figures 1K,L).

### The Effect of Upper Blepharoplasty on Tear Inflammatory Cytokines

Five inflammatory cytokines IL-1β, TNF-α, IL-6, IL-8, and IFN-γ, were examined and compared between subjects with and without dry eye. The baseline levels of IL-6, IL-8, and IFN-γ were significantly higher in subjects with dry eye than in subjects without dry eye (*p* < 0.001, *p* = 0.003, *p* = 0.019) (Figures 2A–C). No significant differences in the preoperative levels of IL-1β and TNF-α were found between the subjects with and without dry eye (Figures 2D,E). Increasing trends of IL-6 and IL-8 were found at 1 (*p* = 0.016, *p* = 0.031) and 3 months (*p* = 0.016, *p* = 0.047) postoperatively in subjects with dry eye (Figures 2A,B). The level of TNF-α significantly increased at 1 month (*p* = 0.031) and returned to the baseline level at 3 months postoperatively in subjects with dry eye (Figure 2E); at 6 months postoperatively, the levels of IL-6 and IL-8 declined slightly but were still higher than the baseline levels (*p* = 0.004, *p* = 0.047) (Figures 2A,B). In subjects without dry eye, only IL-6 significantly increased at 1 and 3 months postoperatively (*p* = 0.008, *p* = 0.016) (Figure 2A). The expression of IL-6, IL-8, and TNF-α returned to baseline levels at 6 months postoperatively (Figures 2A,B,E). No significant fluctuations were found in IL-1β and IFN-γ at the any of the following time (Figures 2C,D).

### The Correlations Between Tear Inflammatory Cytokines and Dry Eye Related Markers

The significantly affected dry eye-related markers were correlated with significant changes in IL-6, IL-8, IFN-γ, and TNF-α levels. The concentration of IL-8 were positively correlated with the OSDI score and meibum expression score (*r* = 0.259, *p* = 0.046; *r* = 0.407, *p* = 0.001 respectively), and negatively correlated with f-NIKBUT (*r* = −0.288, *p* = 0.026) (Figure 3A). The level of TNF-α was positively correlated with the OSDI score (*r* = 0.385, *r* = 0.043), while it was negatively correlated with FBUT (*r* = −0.509, *p* = 0.006) (Figure 3B). However, the levels of IL-6 and IFN-γ were not significantly correlated with any of the dry eye-related markers (Figures 3C,D).

## Discussion

Damage to ocular surface homeostasis following upper blepharoplasty contributes to the development of non-infective postoperative complications, such as dry eye. The emergence of dry eye or worsening of preexisting dry eye may occur in patients with upper blepharoplasty (5). Although usually mild and self-limited, a small fraction of postoperative dry eye might have a course longer than 1 month, even 3 months, according to previous studies (3–5, 22).

In this study, we demonstrated that aggravating symptoms and worse tear film stability induced by upper blepharoplasty were more dramatic and persisted for more than 6 months in patients with preexisting dry eye, whereas those changes were always mild and resolved within 3 months in patients without dry eye. This was consistent with the report of Prischmann et al. (3) that people with preexisting dry eye history were at a higher risk for generating dry eye following cosmetic blepharoplasty. Similar results were reported by Park et al. (23) in cataract surgery in which patient with preexisting dry eye had a higher OSDI score and a lower tear break-up time than those without dry eye after surgery. An explanation for this phenomenon is that the lowered sensory nerve thresholds of the ocular surface generally exist in people with preexisting dry eye, rendering them more susceptible to surgical irritation (24). It is noteworthy that a vicious circle exists in corneal sensation and tear film instability that might aggravate each other to promote the development of dry eye (24, 25).

In addition to unstable tear film, dry eye was characterized by ocular surface inflammation. Proinflammatory cytokines IL-1, IL-6, IL-8, and HLA-DR activators TNF-α and IFN-γ were shown to be strongly correlated with dry eye markers, leading to ocular surface damage and goblet cell reduction (26). Surgical procedures might induce the release of ocular inflammatory cytokines, promoting the development of postoperative dry eye. Li et al. (17) demonstrated that IL-1β, IL-6, IL-8, TNF-α, and IL-17A in conjunctival epithelial cells increased dramatically during the early postoperative period of frontalis suspension surgery. Park et al. (23) showed significant elevations of IL-1β, IL-6, IL-8, macrophage chemoattractant protein-1, TNF-α, and IFN-γ in lacrimal tears after cataract surgery. Zhang et al. (15) compared the levels of NGF, TGF-β1, IL-1α, and TNF-α in tears after femtosecond lenticule extraction and LASIK, and found increasing trends in both surgeries. In our study, similar increasing trends were found in the levels of IL-6, IL-8, and TNF-α following upper blepharoplasty. Positive correlations were found in our study between symptoms and inflammatory cytokines (IL-8 and TNF-α), which agreed with the correlations reported by Li et al. (17) and Zhang et al. (15). TNF-α is secreted by macrophages, binding to TNF receptor1 (TNFR1), and TNF receptor2 (TNFR2) to trigger several inflammatory signaling pathways such as NF-κB signals (27). IL-8 is an important member of IL family, showing strong attractive chemotactic effects to T cells and neutrophil cells (28). Amplification of whether TNF-α or IL-8 might lead to the damage of lacrimal gland and meibomian gland. Iatrogenic injury of the eyelid skin and orbicularis oculi muscle in upper blepharoplasty is likely to induce lymphocyte accumulation and the release of inflammatory cytokines, activating the inflammation cascade. Each of these inflammatory cytokines potentially contributes to the activation of sensory nerve terminals by directly inducing ongoing nerve activity or reducing sensory nerve thresholds (24). Therefore, the increased irritation following upper blepharoplasty is partially attributed to an increased inflammation level, except for tear film instability.

Lam et al. (29) showed that meibomian gland dysfunction is associated with increased levels of IL-6, IL-8, TNF-α, and IFN-γ. In this study, we found that the levels of IL-8 and TNF-α were negatively correlated with tear film break-up time, and IL-8 was negatively correlated with meibum quality and expressibility, which is in accordance with the results of Park et al. (23) reported after cataract surgery. These results verified that worse meibomian gland function was associated with a higher level of inflammation (29). Upper blepharoplasty-induced inflammation may lead to meibomian gland obstruction and abnormal meibum release. Moreover, persistent chemosis due to postoperative inflammation might destroy the goblet cells and lead to an unstable tear film (3, 4).

It is generally considered that cosmetic blepharoplasty, especially trimming the orbicularis oculi muscle, might change the dynamics of eyelid closure, tear pumping, and tear distribution, causing a decreased blink rate and tear production, and increased tear evaporation (5, 13, 30). Kim et al. (31) found a significant increase in the Schirmer test at 1 month postoperatively, while Watanabe et al. (32) showed a decrease in tear volume after cosmetic blepharoplasty. However, no significant changes in tear production, including the Schirmer I test and TMH, were observed in our study. In addition, we found that eyelid dynamics represented by blink frequency and an incomplete blink rate were not significantly changed by eyelid surgery, which is consistent with the study performed by Mak et al. (14). They proposed that blink dynamics did not change after upper blepharoplasty and were unlikely to be the cause of dry eye following this surgery (14). Although mechanical alteration of the corneoscleral and conjunctival interface in eyelid surgery was considered sufficient to aggravate or unmask a subclinical condition, our study found that excision of a small portion of the preseptal orbicularis oculi muscle did not obviously affect the lacrimal pump function and blink patterns.

There are several limitations of this study including the relatively small number of subjects and inadequate analysis of inflammatory cytokines. In addition, no evaluation was conducted on the effects of perioperative agents, such as anti-inflammatory agents. Postoperative management is challenging and should be discussed preoperatively. Thus, further studies should be conducted with a longer follow-up period, a larger number of subjects with and without anti-inflammatory agents and more comprehensive examination and analysis of inflammatory cytokines to illustrate the definite role of inflammation in the postoperative dry eye, and to investigate the appropriate management of patients undergoing cosmetic blepharoplasty.

To the best of our knowledge, this study is the first to identify that ocular surface inflammation induced by upper blepharoplasty plays a critical role in the development of postoperative dry eye, and is closely related to irritation symptoms, tear film instability, and meibomian gland dysfunction. Furthermore, preexisting dry eye is considered a higher risk factor to generate worse and persistent ocular surface damage. It is necessary for surgeons to complete a preoperative assessment, recognize preoperative dry eye, and address rational drug use such as artificial tears and topical anti-inflammatory agents during the perioperative period. It is beneficial for patients undergoing upper blepharoplasty to reduce postoperative dry eye and improve patient satisfaction and quality of life. Moreover, to improve the preoperative assessment, elevated inflammatory cytokines might be a more sensitive marker for the development of dry eye than traditional parameters, especially in those with separated symptoms and signs.

## Data Availability Statement

The original contributions presented in the study are included in the article/Supplementary Material, further inquiries can be directed to the corresponding author/s.

## Ethics Statement

The studies involving human participants were reviewed and approved by Ethics Committee of the Eye, Ear, Nose and Throat Hospital of Fudan University (2019093-1). The patients/participants provided their written informed consent to participate in this study.

## Author Contributions

LG, NS, and SZ: conception, design, writing, review, and revision of the manuscript. LG and SZ: development of methodology. NS: surgical operation. SZ: acquisition of data (patients follow up and examination) and analysis and interpretation of data. All authors contributed to the article and approved the submitted version.

## Funding

This work was supported by the Natural Science Foundation of China (NSFC) (82070924) and the Foundation of Shanghai Minhang District Health Commission (2018MW61).

## Conflict of Interest

The authors declare that the research was conducted in the absence of any commercial or financial relationships that could be construed as a potential conflict of interest.

## Publisher's Note

All claims expressed in this article are solely those of the authors and do not necessarily represent those of their affiliated organizations, or those of the publisher, the editors and the reviewers. Any product that may be evaluated in this article, or claim that may be made by its manufacturer, is not guaranteed or endorsed by the publisher.

## Abbreviations

OSDI: Ocular Surface Disease Index
TMH: tear meniscus height
NIKBUT: non-invasive tear break-up time
f-NIKBUT: first non-invasive tear break-up time
av-NIKBUT: average non-invasive tear break-up time
FBUT: fluorescein tear film break-up time
FL: fluorescein staining
IFN-γ: interferon-gamma
IL: interleukin
LLT: lipid layer thickness
TNF-α: tumor necrosis factor alpha.
